# The strategic role of CSP to disseminate knowledge in the field of food and nutrition

**DOI:** 10.1590/0102-311XEN129724

**Published:** 2024-08-19

**Authors:** Inês Rugani Ribeiro de Castro, Gilberto Kac

**Affiliations:** 1 Instituto de Nutrição, Universidade do Estado do Rio de Janeiro, Rio de Janeiro, Brasil.; 2 Instituto de Nutrição Josué de Castro, Universidade Federal do Rio de Janeiro, Rio de Janeiro, Brasil.

The field of food and nutrition has undergone major transformations in Brazil and worldwide in the 40 years of the existence of *Cadernos de Saúde Pública* (CSP). In Brazil, this period is marked by profound epidemiological changes: from hunger, malnutrition, and nutritional deficiencies as the only priority on the public health agenda, to the midst of obesity and food-related chronic non-communicable diseases, and, more recently, to a scenario of increased hunger and food and nutritional insecurity concomitant with the increase in obesity rates ^1^ ^,^ ^2^ ^,^ ^3^. This period also marks important transformations regarding the institutionalization of food and nutrition policies, with rearrangements in the arenas and instances of governance, as well as the strengthening of the food and nutrition area in the Brazilian Unified National Health System (SUS) and the creation of Brazilian National System for Food and Nutrition Security (SISAN), of an intersectoral nature, which brings new opportunities and challenges to advance the agenda of these public policies ^4^. Another relevant fact of this period was the expansion of graduate education in nutritional sciences, for which an important inflection point was the creation of the National Forum of Coordinators of Postgraduate Programs in Food and Nutrition in 2006 ^5^ and, later, of the area of Nutrition at the Brazilian Coordination for the Improvement of Higher Education Personnel (CAPES), in 2011 ^6^, and the continuous expansion of the number of *stricto sensu* graduate courses in this area, in addition to the expansion of master’s and doctoral programs in Public Health, several of which have research groups and disciplines in food and nutrition.

In this Editorial, we reflect on how this journal has been sensitive to the transformative milestones of food and nutrition. It was structured, since its inception, in a space that expresses and dialogues with the described process of change, as well as it disseminates academic production on this theme with different perspectives (such as Epidemiology; Social Sciences and Humanities; and Politics, Planning and Management) and, at the same time, contributes to advancing and directing knowledge production and public policies in this area. To illustrate, we chose to highlight some addressed topics, publications, and thematic issues that express this trajectory. We do not intend to carry out a detailed and rigorous study on how the theme has been addressed daily in the pages of CSP. Our intention, with this reflection, is to record, as in an informal conversation, moments that we consider remarkable. Before embarking on this journey, we consider important to note a significant change in CSP in 2001, which was the inclusion of a food and nutrition dedicated section. Therefrom, we present this reflection: Kac was CSP Associate Editor for food and nutrition from 2001 to 2015 and Castro took over this role ever since.

Reflecting a structural aspect of our society, the themes of hunger and, more recently, food (in)security, have been addressed by different authors over these 40 years, whether in their interface with the population epidemiological profile, or from the determinants perspective, or even regarding public policies. As an illustration, some of the essays by Arruda ^7^, Arruda & Figueira ^8^ and Batista Filho ^9^, published over the first 20 years of CSP, and editorials that discussed the dismantle of the Brazilian National Food and Nutrition Security Council in 2018 ^10^ as well as its resettlement, in 2023 ^11^. In dialogue with this theme, some publications refer to the nutritional status of different population groups and their temporal trend. An example is the thematic issue published in 1993 (vol. 9 Suppl 1) that focused on physical growth and development, approached from different perspectives.

Several very relevant topics on the food and nutrition agenda have also been addressed in other thematic issues published by CSP. In 2003, when the issue of obesity was gaining prominence on the health agenda in low- and middle-income countries, the thematic issue *The Nutritional Transition and the Epidemiology of Obesity in Latin America* (vol. 19 Suppl 1) was published. It comprises articles aiming to contribute to a better understanding of the dynamics of the nutrition transition phenomenon and, above all, of the role that obesity played in the early 2000s, in the complex epidemiological profile of different population groups in countries as Peru, Chile, Argentina, Uruguay, and Brazil. These studies confirmed the increase in obesity in children, adolescents, adults, and women of reproductive age, as well as pointed to a sedentary lifestyle and the intake of inadequate diets as the main determinants, and above all, called for a greater diversity of public policies to encompass effective actions for obesity prevention and control.

In 2008, the thematic issue *Maternal and Childhood Nutritional Epidemiology and the Agenda for Health Research Priorities in Brazil* (vol. 24 Suppl 2) was published. The objective of this was to disseminate relevant knowledge produced in the context of the projects funded by the Call for Research Projects on Food and Nutrition, launched in 2004 by the Brazilian National Research Council (CNPq) and by the then newly created Department of Science and Technology of the Brazilian Ministry of Health, which aimed to reduce the gap between the production of scientific knowledge, the population health needs, and policies formulation. The execution of this call for research was one of the government’s successful initiatives in financing specific areas of knowledge, consistent with what was established in the Brazilian National Agenda of Priorities in Health Research. This publication once again highlights the concern and commitment of CSP to the agenda of the food and nutrition field.

In 2021, in a context of dismantling public policies that guaranteed rights, CSP welcomed the proposal of the General-Coordination of Food and Nutrition of the Brazilian Ministry of Health, in partnership with the Pan American Health Organization (PAHO), to prepare a thematic issue in celebration of the 20th anniversary of the Brazilian National Food and Nutrition Policy (PNAN) (vol. 37 Suppl 1). This publication brings together important reflections and evidence on this policy trajectory and the questions it must answer. It also points out the challenges of the current scenario for its full implementation and strengthening. Among the various topics covered are: the PNAN in primary health care, the promotion of adequate and healthy eating, the evolution of food and nutrition regulatory measures, as well as conflicts of interest and strategies of corporate political action in the definition of food and nutrition policies.

CSP also published thematic issues with results from national household-based surveys, such as the *Brazilian National Health Survey* (PNS 2019) (vol. 38 Suppl 1) and the *Brazilian National Survey on Child Nutrition* (ENANI 2019) (vol. 39 Suppl 2), which relate to the theme of food and nutrition. The first thematic issue presents the situation of the main chronic non-communicable diseases and the ways of life of the Brazilian population, including information on their anthropometric nutritional status and food consumption practices. The second addresses in detail the survey that update indicators of the nutritional status of children under 5 years of age, aiming to support the reformulation of policies in food and nutrition, after 13 years without national data on this topic. This special issue brought relevant data from the first nationwide and household-based study to deal in depth with some themes of childhood food and nutrition, such as food consumption (measured by the 24-hour recall method), micronutrient deficiencies (measured by 12 biomarkers), and practices related to breastfeeding, such as cross-breastfeeding.

Finally, we would like to mention the publication of the article *A New Classification of Foods Based on the Extent and Purpose of their Processing* in CSP ^12^. It is a seminal article on the NOVA Classification of foods: the second article on this topic, published by the group of researchers who conceived this classification, and the first to articulate the classification with an empirical basis. NOVA represents a new paradigm for the production of knowledge about food and dietary intake. When classifying (in its current version) ^13^ food in four groups according to their processing extent and purpose, and conceiving the group of “ultra-processed foods”, this proposal offered a totally innovative data analysis perspective of dietary intake, which has proven to be very useful for the production of robust evidence on the association between this dieatry intake and negative health outcomes ^14^. It has also been adopted in documents that induce public policies in Brazil and other countries ^15^ ^,^ ^16^ ^,^ ^17^.

As demonstrated in this brief report, CSP has been a strategic space for the debate on food and nutrition and for the dissemination of relevant knowledge to support public policies to positively impact the living and health conditions of populations.

The process of profound transformations mentioned at the beginning of this Editorial is still underway, which presents new challenges to the food and nutrition agenda, such as the relations between food systems and the climate crisis; the corporate capture of governance spaces for public policy, among others. These challenges are added to unresolved issues such as hunger, as well as race, gender, and social class inequalities and threats to socio-biodiversity and food cultures.

We hope that CSP continues to fulfill its fundamental role for advancing the knowledge in food and nutrition and for political advocacy in this field.

Long and prosperous life for CSP!
