# Supplementary material for: The strategic role of CSP to disseminate knowledge in the field of food and nutrition
Source: Cad Saude Publica. 2024 Aug 19;40(8):e00129724. doi: 10.1590/0102-311XEN129724 (PMC11338594; doi:10.1590/0102-311XEN129724)
Supplement: Translation

# Translation: O papel estratégico de CSP na disseminação de conhecimento no campo da alimentação e nutrição

**DOI:** 10.1590/0102-311XPT129724

**Authors:** Inês Rugani Ribeiro de Castro, Gilberto Kac

**Affiliations:** 1 Instituto de Nutrição, Universidade do Estado do Rio de Janeiro, Rio de Janeiro, Brasil.; 2 Instituto de Nutrição Josué de Castro, Universidade Federal do Rio de Janeiro, Rio de Janeiro, Brasil.

O campo da alimentação e nutrição sofreu grandes transformações no Brasil e no mundo nos 40 anos de existência de *Cadernos de Saúde Pública* (CSP). No Brasil, esse período é marcado por profundas mudanças epidemiológicas: da fome, desnutrição e deficiências nutricionais como única prioridade na agenda da Saúde Coletiva para a centralidade da obesidade e das doenças crônicas não transmissíveis relacionadas à alimentação, e, mais recentemente, para um cenário de recrudescimento da fome e da insegurança alimentar e nutricional concomitante com o aumento da obesidade ^1^ ^,^ ^2^ ^,^ ^3^. Também marcam esse período importantes transformações em relação à institucionalidade das políticas de alimentação e nutrição, com rearranjos nas arenas e instâncias de governança, fortalecimento da área de alimentação e nutrição no Sistema Único de Saúde (SUS) e a criação do Sistema de Segurança Alimentar e Nutricional (SISAN), de natureza intersetorial, que apresenta novas oportunidades e desafios para fazer avançar a agenda dessas políticas públicas ^4^. Outro fato relevante desse período foi a expansão da formação pós graduada em ciências nutricionais, que tem como importante ponto de inflexão a criação do Fórum Nacional de Coordenadores de Programas de Alimentação e Nutrição em 2006 ^5^ e, posteriormente, da área de Nutrição na Coordenação de Aperfeiçoamento de Pessoal de Nível Superior (CAPES), em 2011 ^6^, e a contínua expansão do número de cursos de pós-graduação *stricto sensu* nessa área, além da ampliação de cursos de mestrado e doutorado em Saúde Coletiva, muitos dos quais com grupos de pesquisa e disciplinas em alimentação e nutrição.

Neste Editorial, refletimos sobre o quanto esta revista, tão importante para a Saúde Coletiva, tem sido sensível aos marcos transformativos da alimentação e nutrição, configurando-se desde seu início como espaço que expressa e dialoga com o processo de mudança acima descrito, difunde a produção acadêmica sobre essa temática com diferentes recortes (como os da Epidemiologia, das Ciências Sociais e Humanas, e de Política, Planejamento e Gestão) e, ao mesmo tempo, contribui para os avanços e rumos da produção do conhecimento e das políticas públicas nessa área. Para ilustrar esse compromisso, optamos por destacar alguns temas abordados, publicações e números temáticos que expressam essa trajetória. Não temos a pretensão de realizar um estudo detalhado e rigoroso sobre como essa temática vem sendo abordada cotidianamente nas páginas de CSP. Nossa intenção, com esta reflexão, é registrar, como em uma conversa informal, *flashes* que consideramos marcantes nesse percurso. Antes de embarcarmos nessa jornada, consideramos importante registrar uma mudança marcante ocorrida em 2001 em CSP, que foi a inclusão de uma editoria dedicada à alimentação e nutrição. É desse lugar que apresentamos essa reflexão: Kac atuou como Editor Associado de CSP para alimentação e nutrição de 2001 a 2015, e Castro está nessa função desde então.

Refletindo um aspecto estrutural de nossa sociedade, os temas da fome e, mais recentemente, da (in)segurança alimentar têm sido pautados por diferentes autores ao longo desses 40 anos, seja em sua interface com o perfil epidemiológico da população, seja na perspectiva de seus determinantes ou, ainda, de políticas públicas que respondam a essas mazelas. Exemplos disso são alguns dos ensaios de Arruda ^7^, Arruda & Figueira ^8^ e Batista Filho ^9^, publicados ao longo dos primeiros 20 anos de CSP, e editoriais que discutiram a extinção do Conselho Nacional de Segurança Alimentar e Nutricional em 2018 ^10^, e sua reinstalação em 2023 ^11^. 

Em diálogo com essa temática estão as publicações referentes ao estado nutricional de diferentes grupos populacionais e sua tendência temporal. O número temático publicado em 1993 (vol. 9 Suppl 1), que teve como foco o crescimento e o desenvolvimento físicos, abordados sob diferentes perspectivas, é exemplo disso.

Diversos temas relevantes na agenda de alimentação e nutrição também vêm sendo abordados em outros números temáticos oportunamente publicados por CSP. Em 2003, quando a questão da obesidade ganhava centralidade na agenda da saúde em países de baixa e média renda, foi publicado o número temático *A Transição Nutricional e a Epidemiologia Nutricional na América Latina* (vol. 19 Suppl 1), contemplando artigos que visavam colaborar para o melhor entendimento da dinâmica do fenômeno de transição nutricional e, sobretudo, do papel que a obesidade exercia, no começo dos anos 2000, no complexo perfil epidemiológico em distintos grupos populacionais de países tão diferentes como Peru, Chile, Argentina, Uruguai e Brasil. Esses estudos confirmaram o aumento da obesidade em crianças, adolescentes, adultos e mulheres em idade reprodutiva, além de apontar o sedentarismo e o consumo de dietas inadequadas como os principais determinantes, e, mais que tudo, clamaram por maior diversidade de políticas públicas que abarcassem ações efetivas de prevenção e controle da obesidade.

Em 2008, ocorreu a publicação do suplemento *Epidemiologia Nutricional Materno-infantil e a Agenda de Prioridades de Pesquisa em Saúde no Brasil* (vol. 24 Suppl 2). O objetivo desse número temático foi divulgar conhecimentos relevantes produzidos no bojo dos projetos financiados pelo edital sobre Alimentação e Nutrição, lançado em 2004 pelo Conselho Nacional de Desenvolvimento Científico e Tecnológico (CNPq) e pelo então recém-criado Departamento de Ciência e Tecnologia do Ministério da Saúde, que visava reduzir a distância entre a produção do conhecimento científico, as reais necessidades de saúde da população e a formulação de políticas. A execução desse edital configurou-se em uma das iniciativas bem sucedidas do governo em financiar áreas específicas do conhecimento coerentes com o estabelecido na Agenda Nacional de Prioridades de Pesquisa em Saúde. Essa publicação evidencia mais uma vez o acolhimento e o compromisso de CSP com as pautas do campo da alimentação e nutrição.

Em 2021, em um contexto de desmonte de políticas públicas de garantia de direitos, CSP acolheu a proposta da Coordenação-Geral de Alimentação e Nutrição do Ministério da Saúde, em parceria com a Organização Pan-Americana da Saúde (OPAS), de elaborar um número temático em celebração aos 20 anos da Política Nacional de Alimentação e Nutrição (PNAN) (vol. 37 Suppl 1). Esta publicação reúne importantes reflexões e evidências sobre a trajetória dessa política e questões que ela deve responder. Aponta, também, os desafios do cenário atual para sua plena implementação e fortalecimento. Entre os vários temas abordados, estão: a PNAN na atenção primária à saúde, a promoção da alimentação adequada e saudável, a evolução das medidas regulatórias em alimentação e nutrição e os conflitos de interesse e estratégias de ação política corporativa na definição de políticas de alimentação e nutrição.

CSP também publicou números temáticos com resultados de inquéritos nacionais de base domiciliar, como a *Pesquisa Nacional de Saúde* (PNS 2019) (vol. 38 Suppl 1) e o *Estudo Nacional de Alimentação e Nutrição Infantil* (ENANI 2019) (vol. 39 Suppl 2), que se relacionam com a temática de alimentação e nutrição. O primeiro número temático apresenta a situação das principais doenças crônicas não transmissíveis e dos modos de vida da população brasileira, e inclui informações sobre seu estado nutricional antropométrico e suas práticas de consumo alimentar. O segundo aborda de forma detalhada o inquérito que objetivou atualizar indicadores do estado nutricional de crianças menores de 5 anos, visando subsidiar a reformulação de políticas na área de alimentação e nutrição depois de 13 anos sem dados nacionais sobre essa temática. Esse número especial contém dados relevantes desse que é o primeiro estudo de abrangência nacional e de base domiciliar a tratar com profundidade alguns temas de alimentação e nutrição na infância, como o consumo alimentar (aferido pelo método de recordatório de 24 horas), deficiências de micronutrientes (aferidas por meio de 12 biomarcadores) e práticas relacionadas à amamentação, como a amamentação cruzada.

Por fim, gostaríamos de citar a publicação do artigo *A New Classification of Foods Based on the Extent and Purpose of Their Processing* [*Uma Nova Classificação de Alimentos Baseada na Extensão e Propósito do seu Processamento*] em CSP ^12^. Trata-se de um artigo seminal sobre a Classificação NOVA de alimentos: o segundo artigo sobre esse tema publicado pelo grupo de pesquisadores que concebeu essa classificação e o primeiro a articular a classificação com uma base empírica. A NOVA representa um novo paradigma para produção do conhecimento sobre alimentação e consumo alimentar. Ao classificar (em sua atual versão) ^13^ os alimentos em quatro grupos de acordo com a extensão e o propósito de seu processamento e conceber o grupo de “alimentos ultraprocessados”, essa proposta ofereceu uma perspectiva de análise de dados de consumo alimentar totalmente inovadora, que tem se mostrado muito útil para a produção de evidências robustas sobre a associação entre o consumo desses alimentos e desfechos negativos em saúde ^14^. Ela também tem sido adotada em documentos indutores de políticas públicas no Brasil e em outros países ^15^ ^,^ ^16^ ^,^ ^17^.

Como se pode depreender desse breve relato, CSP tem sido um espaço estratégico para o debate sobre alimentação e nutrição e para a difusão de conhecimento relevante para subsidiar políticas públicas que impactem positivamente as condições de vida e de saúde das populações.

Cabe dizer que segue em curso o processo de profundas transformações mencionado no início deste Editorial, que apresenta novos desafios para a agenda de alimentação e nutrição, como a relação entre sistemas alimentares e a crise climática, a captura corporativa dos espaços de governança de políticas públicas, entre outros. Esses desafios se somam a questões não superadas, como a fome, as desigualdades de raça, gênero e classe social, e as ameaças à sociobiodiversidade e às culturas alimentares.

Desejamos que CSP continue cumprindo seu papel fundamental para o avanço do conhecimento em alimentação e nutrição e para a incidência política nesse campo.

Vida longa e pujante para CSP!

# Translation: El papel estratégico de CSP para difundir el conocimiento en el campo de la alimentación y la nutrición

**DOI:** 10.1590/0102-311XES129724

**Authors:** Inês Rugani Ribeiro de Castro, Gilberto Kac

**Affiliations:** 1 Instituto de Nutrição, Universidade do Estado do Rio de Janeiro, Rio de Janeiro, Brasil.; 2 Instituto de Nutrição Josué de Castro, Universidade Federal do Rio de Janeiro, Rio de Janeiro, Brasil.

El campo de la alimentación y la nutrición ha experimentado grandes transformaciones en Brasil y en otros países en los 40 años de existencia de los *Cadernos de Saúde Pública* (CSP). Este período marcó profundos cambios epidemiológicos en Brasil, como el hambre, la desnutrición y las deficiencias nutricionales como prioridades en la agenda de salud pública, pasando por la obesidad y las enfermedades crónicas no transmisibles relacionadas con la alimentación, y, más recientemente, por un empeoramiento del hambre y la inseguridad alimentaria y nutricional, sumado al aumento de la obesidad ^1^ ^,^ ^2^ ^,^ ^3^. Además, hubo importantes transformaciones respecto a la institucionalidad de las políticas de alimentación y la nutrición, con reordenamientos en el ámbito gubernamental, fortalecimiento del campo de alimentación y la nutrición en el Sistema Único de Salud brasileño (SUS) y la creación del Sistema de Seguridad Alimentaria y Nutricional (SISAN), de carácter intersectorial, que aporta con nuevas oportunidades y desafíos en la agenda de estas políticas públicas ^4^. Otro hecho relevante fue la ampliación de la oferta de posgrado en Ciencias Nutricionales -que motivó la creación del Foro Nacional de Coordinadores de Programas de Alimentación y Nutrición en 2006 ^5^ y, posteriormente, del área de Nutrición en la Coordinación de Perfeccionamiento de Personal de Nivel Superior (CAPES) en 2011 ^6^, la continua expansión de los estudios de posgrado *stricto sensu* en este campo y el incremento de la oferta de la maestría y doctorado en Salud Colectiva, con varios grupos de investigación y asignaturas de alimentación y la nutrición.

En este Editorial se presenta cómo esta revista tan significativa para la Salud Colectiva ha sido sensible a los hitos transformadores de alimentación y la nutrición como un espacio de expresión y diálogo con los cambios mencionados para difundir la producción académica desde variados recortes (como Epidemiológico; de Ciencias Sociales y Humanas; y de la Política, Planificación y Gestión) y, al mismo tiempo, contribuye a los avances y caminos de la producción de conocimiento y políticas públicas en esta materia. Para ilustrar este compromiso, podemos destacar algunos temas abordados en publicaciones y ediciones temáticas de CSP. No pretendemos realizar un estudio detallado y riguroso sobre cómo CSP ha abordado la temática en sus páginas. Nuestra intención con esta reflexión es registrar, como en una conversación, algunos “momentos” que consideramos notables en este recorrido. Antes de empezar, consideramos importante destacar el cambio que ocurrió en 2001 en CSP: la inclusión del departamento editorial dedicado a alimentación y la nutrición. Es desde este lugar que hacemos esta reflexión: Kac fue el Editor Asociado de CSP en alimentación y la nutrición de 2001 a 2015 y, desde entonces, Castro está en este puesto.

Los problemas estructurales de nuestra sociedad como el hambre y, más recientemente, la (in)seguridad alimentaria han sido objeto de estudios de diversos autores a lo largo de estos 40 años, ya sea en interfaz con el perfil epidemiológico de la población, o desde la perspectiva de los determinantes o incluso de las políticas públicas que buscan dar una respuesta a esta problemática. Ejemplos de esto son algunos de los ensayos de Arruda ^7^ Arruda & Figueira ^8^ y Batista Filho ^9^, publicados durante los primeros 20 años de CSP, y editoriales que discutieron la extinción del Consejo Nacional de Seguridad Alimentaria y Nutricional en 2018 ^10^ y su reinstalación en 2023 ^11^.

En diálogo con esta temática están las publicaciones sobre el estado nutricional de diversos grupos de población y su tendencia temporal. Esto ha sido tratado, por ejemplo, en el número temático de 1993 (vol. 9 Suppl 1) que se centró en el crecimiento y desarrollo físico desde diferentes perspectivas.

Otras ediciones temáticas de CSP también abordaron temas muy relevantes para la agenda de alimentación y la nutrición. En 2003 cuando el tema de la obesidad ganó centralidad en la agenda de salud en los países de bajo y medio desarrollo, se publicó la edición temática *A Transição Nutricional e a Epidemiologia Nutricional na América Latina* [*La Transición Nutricional y la Epidemiología Nutricional en América Latina*] (vol. 19 Suppl 1), cuyos artículos pretendían dar una mejor comprensión de la dinámica del fenómeno de transición nutricional y, sobre todo, del papel que la obesidad jugaba a principios de los años 2000 en un complejo perfil epidemiológico em diferentes grupos poblacionales de países tan distintos como Perú, Chile, Argentina, Uruguay y Brasil. Los estudios constataron un aumento de la obesidad en niños, adolescentes, adultos y mujeres en edad reproductiva como un reflejo del estilo de vida sedentario y del consumo de dieta inadecuada, además, llamaron la atención a la necesidad de implementar más políticas públicas con acciones efectivas de prevención y control de la obesidad.

En 2008 se publicó el número temático *Epidemiologia Nutricional Materno-infantil e a Agenda de Prioridades de Pesquisa em Saúde no Brasil* [*Epidemiología Nutricional Materno-infantil y la Agenda de Prioridades de Investigación en Salud en Brasil*] (vol. 24 Suppl 2). Esta edición temática buscaba difundir los estudios que recibieron apoyo en el marco de la Convocatoria sobre Alimentación y Nutrición, que había sido lanzada en 2004 por el Consejo Nacional de Desarrollo Científico y Tecnológico (CNPq) y por el entonces recién creado Departamento de Ciencia y Tecnología del Ministerio de Salud brasileño, cuyo objetivo era reducir la brecha existente entre la producción de conocimientos científicos, las necesidades reales de salud de la población y la formulación de políticas públicas. La ejecución de esta convocatoria fue una de las iniciativas exitosas del gobierno para financiar áreas específicas de conocimiento consistentes con lo establecido por la Agenda Nacional de Prioridades de Investigación en Salud. Esta publicación muestra, una vez más, la aceptación y el compromiso de CSP con las agendas del campo de alimentación y la nutrición.

En 2021, frente a un contexto de desmonte de las políticas públicas de garantía de derechos, CSP aceptó la propuesta de la Coordinación General de Alimentación y Nutrición del Ministerio de Salud, en colaboración con la Organización Panamericana de la Salud (OPS), para elaborar una edición temática en celebración de los 20 años de la Política Nacional de Alimentación y Nutrición (PNAN) (vol. 37 Suppl 1). Esta publicación reúne importantes reflexiones y evidencias sobre la trayectoria de esta política y cuestiones a las cuales debe responder. También señala los desafíos del escenario actual para su plena implementación y fortalecimiento. Entre los diversos temas tratadosse encuentran: la PNAN en atención primaria de salud, la promoción de una alimentación adecuada y saludable, la evolución del marco regulatorio en alimentación y la nutrición y los conflictos de interés y las estrategias de acción política corporativa en la definición de políticas alimentarias y nutricionales.

CSP también publicó ediciones temáticas con los resultados de encuestas nacionales de hogares, como la *Encuesta Nacional de Salud* (PNS-2019) (vol. 38 Suppl 1) y el *Estudio Nacional sobre Alimentación y Nutrición Infantil* (ENANI-2019) (vol. 39 Suppl 2), que se relacionan con el tema de alimentación y la nutrición. En la primera se expone la situación de las principales enfermedades crónicas no transmisibles y modos de vida de la población brasileña, además de incluir información sobre su estado nutricional antropométrico y sus prácticas de consumo de alimentos. En la segunda se aborda en detalle la encuesta que actualizó los indicadores del estado nutricional de niños menores de 5 años de edad para fundamentar la reformulación de políticas en el campo de alimentación y la nutrición después de 13 años sin datos nacionales sobre el tema. Esta edición especial planteó datos relevantes del primer estudio nacional domiciliar al tratar en profundidad algunos temas de alimentación y la nutrición en la infancia, como el consumo de alimentos (medido por el método de recuerdo de 24 horas), las deficiencias de micronutrientes (estimadas con 12 biomarcadores) y las prácticas relacionadas con la lactancia materna, como la lactancia materna cruzada.

Por último, nos gustaría mencionar el artículo *A New Classification of Foods Based on the Extent and Purpose of Their Processing* [*Una Nueva Classificación de Alimentos Basada en el Alcance y el Propósito de su Procesamiento*] publicado por CSP ^12^. Este es un artículo seminal sobre la Clasificación NOVA de Alimentos: el segundo artículo sobre este tema publicado por el grupo de investigadores que creó esta clasificación, y el primer que la articuló con una base empírica. NOVA representa un nuevo paradigma en la producción de conocimiento sobre la alimentación y el consumo de alimentos. Al clasificar (en su versión actual) ^13^ los alimentos en cuatro grupos según el grado de procesamiento y definir el grupo de “alimentos ultraprocesados”, esta propuesta presenta una perspectiva de análisis de datos de consumo de alimentos totalmente innovadora, que ha sido muy útil para producir evidencia sólida sobre la asociación entre el consumo de alimentos y los desenlaces negativos para la salud ^14^. Además, esta perspectiva ha sido adoptada en documentos de promoción de políticas públicas en Brasil y otros países ^15^ ^,^ ^16^ ^,^ ^17^.

En este breve Editorial se puede apreciar que CSP ha sido un espacio estratégico de debate sobre alimentación y la nutrición y de difusión de conocimientos relevantes para la formulación de políticas públicas que impacten positivamente en la situación de vida y salud de las poblaciones.

Cabe agregar que el proceso de profundas transformaciones mencionado al inicio de este editorial se encuentra aún en curso. Esto implica nuevos desafíos a la agenda de alimentación y la nutrición, como la relación entre los sistemas alimentarios y la crisis climática; la captura corporativa de espacios de gobernanza de políticas públicas, entre otros. Estos desafíos se suman a los problemas sin resolver, como el hambre, las desigualdades de raza, género y clase social, las amenazas a la sociobiodiversidad y las culturas alimentarias.

Esperamos que CSP siga cumpliendo su importante papel para el avance del conocimiento en alimentación y nutrición y en la incidencia política en este campo.

¡Larga y próspera vida a CSP!
